# Interaction of Carbamazepine with Herbs, Dietary Supplements, and Food: A Systematic Review

**DOI:** 10.1155/2013/898261

**Published:** 2013-08-19

**Authors:** Sophia Yui Kau Fong, Qiong Gao, Zhong Zuo

**Affiliations:** School of Pharmacy, Faculty of Medicine, The Chinese University of Hong Kong, Shatin, New Territories, Hong Kong

## Abstract

*Background*. Carbamazepine (CBZ) is a first-line antiepileptic drug which may be prone to drug interactions. Systematic review of herb- and food-drug interactions on CBZ is warranted to provide guidance for medical professionals when prescribing CBZ. *Method*. A systematic review was conducted on six English databases and four Chinese databases. *Results*. 196 out of 3179 articles fulfilled inclusion criteria, of which 74 articles were reviewed and 33 herbal products/dietary supplement/food interacting with CBZ were identified. No fatal or severe interactions were documented. The majority of the interactions were pharmacokinetic-based (80%). Traditional Chinese medicine accounted for most of the interactions (*n* = 17), followed by food (*n* = 10), dietary supplements (*n* = 3), and other herbs/botanicals (*n* = 3). Coadministration of 11 and 12 of the studied herbal products/dietary supplement/food significantly decreased or increased the plasma concentrations of CBZ. Regarding pharmacodynamic interaction, Xiao-yao-san, melatonin, and alcohol increased the side effects of CBZ while caffeine lowered the antiepileptic efficacy of CBZ. *Conclusion*. This review provides a comprehensive summary of the documented interactions between CBZ and herbal products/food/dietary supplements which assists healthcare professionals to identify potential herb-drug and food-drug interactions, thereby preventing potential adverse events and improving patients' therapeutic outcomes when prescribing CBZ.

## 1. Background

Introduced in 1960s, carbamazepine (CBZ) remains as one of the most commonly prescribed antiepileptic drugs worldwide and has established efficacy for the treatment of partial seizures, generalized tonic-clonic seizures, trigeminal neuralgia, and bipolar disorders [1–6]. Despite its clinical popularity, CBZ possesses several pharmacokinetic properties which make it prone to interaction with coadministered substances, including drugs, herbal products, and food [7]. CBZ is a potent inducer of CYP450 system and is subject to autoinduction. Its metabolism is exclusively hepatic and catalyzed by various enzymes including CYPs, UGTs, and SULTs [8]. CYP3A4 is the most important enzyme involved in the metabolism of CBZ as it leads to the formation of the active metabolite CBZ 10,11-epoxide, which appears to contribute to the toxicity and efficacy of CBZ [9, 10]. Furthermore, CBZ has a considerably narrow therapeutic index of 2-3 while there is a wide interindividual variation in tolerable doses and blood levels, making therapeutic drug monitoring and slow titration necessary [11, 12]. Many side effects associated with CBZ are concentration-related. Nausea, vertigo, dizziness, and blurred vision are examples of CBZ adverse effects which mostly are mild, transient, and reversible if the dosage is reduced or if initiation of treatment is gradual [13]. Signs of toxicity generally occur at plasma CBZ concentrations in excess of 10 to 12 mg/L, with diplopia, nystagmus, and aplastic anemia being the most characteristic ones [11]. Fatal cases of CBZ overdose were also recorded where patients were manifested with cardiac arrhythmias, abnormal movements, and seizures [14]. The occurrence of CBZ overdose is usually accidental, and in most times it is secondary to the coadministration of other substances [15–20].

Since antiepileptic regimens are normally given on a long-term basis, the opportunity of a clinical significant interaction between CBZ and coadministered substances is considerably high. Herbal medicines, dietary supplements, and food may interact with CBZ pharmacokinetically and/or pharmacodynamically which leads to potential clinical consequences. One of the contributing factors towards increasing incidence of herb-drug interaction is the increased popularity of herbal medicines [21]. According to pharmacoepidemiologic surveys, the percentage of epileptic patients concurrently taking complementary and alternative medicines and antiepileptic drugs is considerably high in both developed and developing regions: United States (39%), Cambodia (36%), United Kingdom (34%), Taiwan (16%), Nigeria, (15%) and India (12%), while more than 60% of them did not inform their physicians [22–27]. In China, integrated medicine is a common practice where Western and traditional Chinese medicines are prescribed concurrently for the treatment of epilepsy [28]. Therefore, the opportunity of patients taking CBZ with herbal/dietary supplements is high, and it is necessary to address the safety issues of such combinational use. 

When making clinical decisions on the use of herbal or dietary supplements, the review article is one of the major information sources for healthcare professionals [29]. In view of this, we tried to identify existing review articles that (1) summarized all the reports and studies on the pharmacokinetic and pharmacodynamic interactions of CBZ with herbs, dietary supplements, and food and (2) provided recommendations on their combinational use. It was found that there is a lack of well-conducted systematic review on CBZ and its herb, food, and dietary supplement interactions. The searching strategy adopted by these reviews is not comprehensive enough to identify all the relevant articles. Most of these reviews use general terms such as “herb-drug interaction” as searching keywords and do not focus on one particular drug (e.g., CBZ) [30–33]. This nondrug-specific searching method may result in missing CBZ-relevant papers if the paper does not contain the phrase of “herb-drug interaction”. Besides, there is no single review that covers the interactions between CBZ and all the three aspects of herb, food, and dietary supplement. A systematic review is warranted to provide guidance for healthcare professions when prescribing and monitoring patients taking CBZ. From the review articles that report herb-drug interactions, we can see that herb-drug interactions are often less systematically documented and less familiar to medical practitioners compared to drug-drug interactions. The nonstandardized naming of herbals with several confusing generic names together with an unfamiliar Latin name may make it difficult for medical professionals to anticipate and monitor herb-drug interaction [34]. Furthermore, most herbal formulas (especially traditional Chinese medicine, TCM) contain multiple herbs, making it often impossible to know which herbs and at what doses they are present. 

In view of all the constraints of the existing literature, a comprehensive systematic review focusing on CBZ and overcoming the mentioned hurdles is warranted for healthcare professionals to make proper decisions. In this current report, we conducted a systematic review on interactions between CBZ and herbs, dietary supplements, and food, summarizing the scientific evidence for such interactions and providing recommendations for the combinational use. In addition to the usual databases (e.g., Medline and Embase), we also included several Chinese databases to identify reports of interactions between CBZ and TCM which are written in Chinese. The aim of this review is to provide a clear and systematic presentation of herb and food interactions with CBZ to alert and provide guidance for medical professionals when prescribing CBZ.

## 2. Materials and Methods

### 2.1. Data Sources and Literature Search

A computer-based search of the following English databases was conducted: AMED (1985–Oct. 2012), CINAHL Plus (1937–Oct. 2012), Cochrane Database of Systematic Reviews (2005–Dec. 2011), CENTRAL (Oct. 2012), Embase (1947–Oct. 2012), Medline (1946–Oct. 2012), and SciFinder Scholar (1907–Oct. 2012). The keyword search terms for carbamazepine (“Carbamazepine”, “Tegretol”, “Tegretol XR”, “G-32883”, “5H-Dibenz[b,f]azepine-5-carboxamide”) were combined, using the combination term AND, with a comprehensive list of keywords and MeSH search terms for herbs, food, and dietary supplements (Table 1). Such search list was refined to include most of the relevant articles. No language restriction was imposed during the search, but non-English articles were included only if they contained an English abstract with sufficient information. As defined by the Dietary Supplement Health and Education Act of 1994 (DSHEA), “dietary supplement” refers to any dietary products containing one or more of the following ingredients: vitamin, mineral, herb or other botanical, amino acid, and a dietary substance for use by man to supplement the diet by increasing the total dietary intake. In the current review, we separated “herb or other botanical” out and categorized this group as “herbs” while the remainings were referred to as “dietary supplement”. The third category is “food” which includes any specific traditional food/fruit products or beverages. Paper containing only a general term of “food” without specifying any particular food item will be excluded. In addition to the English databases, four Chinese databases had been searched, including Chinese BioMedical Literature Database (1978–Oct. 2012), China Journal Net (1915–Oct. 2012), Traditional Chinese Medical Database System (1984–Oct. 2012), and Chinese Medical Academic Conference Database (1994–Oct. 2012). The MeSH headings and keywords used for the search were carbamazepine (Chinese name, Chinese common names, and chemical names) in combination with the Chinese equivalent terms of “interaction”, “Chinese herbal medicines”, and “Chinese and Western medicines” (“jie he”, “xiang hu zuo yong”, and “zong yao”, “zong cao yao”, “zong xi yi”). The bibliographies of every retrieved article were checked for any additional pertinent studies. 

### 2.2. Inclusion Criteria and Data Extraction

The selection of relevant reports and evaluation of article eligibility was carried out by two reviewers independently (Fong and Gao). Articles were considered eligible for evaluation if they contained original data involving herb, food, or dietary supplement interactions with CBZ without restriction for *in vitro* studies, animal studies, clinical studies observational studies, or review articles. Any discrepancies were resolved by a third author (Zuo). All relevant literature fulfilling our inclusion criteria were extracted and complied, except for the interacting pairs that have beneficial effects. 

We grouped the natural products into four categories: TCM, other herb/botanical, vitamin/mineral/amino acid, and food. We categorized the mechanisms for pairs of interactions into three types: pharmacokinetics, pharmacodynamics, and both. In order to standardize the names of the included TCMs, the official compendium Pharmacopoeia of the People's Republic of China 2010 (Chinese Pharmacopoeia) was consulted and their Latin names (for herbs) or Chinese pinyin names (for herbal formulae) were presented.

## 3. Results

### 3.1. Literature Search

**Figure 1:**
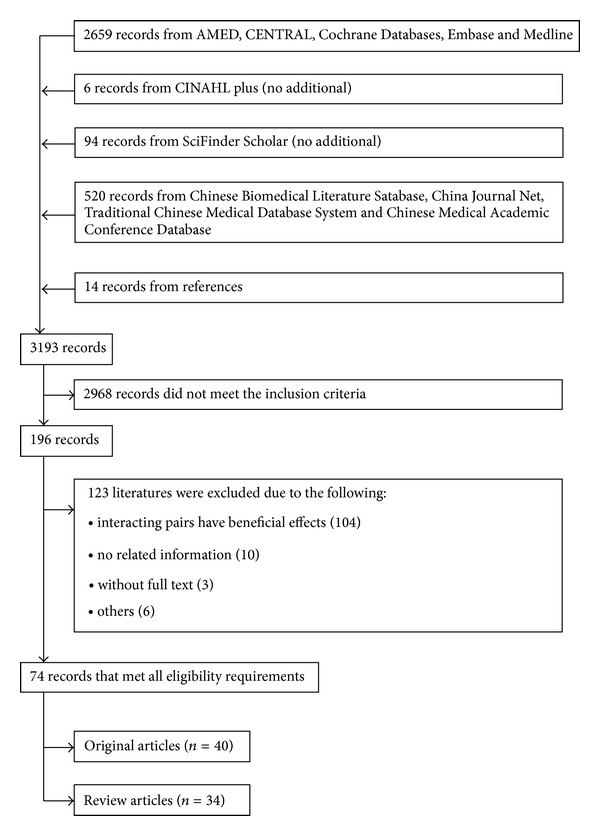
Flow chart of literature search.

A total of 3179 articles was initially found through database searches while an addition of 14 articles were obtained from scrutinizing the bibliographies of relevant literatures. 196 articles fulfilling the inclusion criteria were selected for further evaluation with perfect agreement between the two authors. Finally, seventy-four articles with full text, including 40 original articles and 34 review articles, were qualified to undergo an in-depth review (Figure 1); a total of 33 unique herbal products/dietary supplement/food-CBZ interacting pairs were identified from these articles. Summaries of the *in vitro*, animal, and clinical studies to retrieve information about interactions between CBZ and herbal products/dietary supplement/food for the original studies are listed in Tables 2 and 3, respectively. Among the original studies (*n* = 40), most are animal (*n* = 24) and human (*n* = 14) studies, with 2 mechanistic* in vitro*/*ex vivo* studies. Regarding the studied types of interaction, the majority (*n* = 32) are pharmacokinetic interactions followed by both pharmacokinetic and pharmacodynamic interactions (*n* = 6) and pharmacodynamic interactions (*n* = 2).

### 3.2. Interactions between Herbal Products and CBZ

Nineteen of the included original articles documented the interactions between 20 different herbal products and CBZ, where TCMs in the form of crude drug, extract, or single TCM compound were the major studied herbal products (*n* = 17). Among the 17 documented pharmacokinetic interactions between CBZ and TCMs, *Cassia auriculata* Linn., piperine (an active compound in *Piper longum *Linn.), Platycodonis Radix, and *Polygonum cuspidatum* were demonstrated to increase the plasma level/oral bioavailability of CBZ through decreasing the metabolism of CBZ or improving gastric solubility of CBZ [37, 47–49]. On the other hand, ginkgo biloba, Hu-gan-ning pian, Jia-wei-xiao-yao-san, and Xiao-yao-san decreased the plasma level/oral bioavailbaility of CBZ through increasing the metabolism of CBZ via CYP3A4 induction [40, 44, 53]. Ginsenoside (an active compound in *ginseng*) was also shown to activate CYP3A4 activity *in vitro* and thereby increased CBZ metabolism [39]. Xiao-qing-long-tang and Xiao-cha-hu-tang delayed the time for CBZ to reach peak plasma concentration through decreasing gastric emptying rate [38, 50], whereas Paeoniae Radix decreased the *T*_max⁡_ of CBZ through possibly improving dissolution of CBZ [46].* Acorus calamus* Linn., berberine (an active compound in Coptidis rhizome), *Cardiospermum halicacabum* Linn., Chai-hu-jia-long-gu-mu-li-tang, and *Hypericum perforatum *Linn. did not alter the plasma levels or other pharmacokinetic parameters of CBZ in animal or human studies [35–38, 42]. As regards the pharmacodynamic interactions between CBZ and TCMs, there were three articles reporting the effect of TCMs on the efficacy and/or side effects of CBZ. *Acorus calamus* Linn. was shown to have an additive antiepileptic activity with CBZ in an animal study [35]. Xiao-yao-san might increase the incidence of dizziness, blurred vision, skin rash, and nausea when coadministered with CBZ in a clinical study [53] while *Cardiospermum halicacabum* Linn. and *Cassia auriculata* Linn. did not potentiate CBZ-related toxicity in rats [37]. The three remaining herbal products categorized as “other herb/botanical” that had documented herb-drug interactions with CBZ were Ispaghula husk, mentat, and septilin. Ispaghula husk, also more commonly known as psyllium, decreased the oral bioavailability and absorption of CBZ in four healthy volunteers [43] while septilin also decreased the absorption of CBZ in rabbits possibly through interfering with the gastric emptying or intestinal transit time [50]. On the contrary, mentat (BR 16A) increased the bioavailability of CBZ in rabbits through an unknown mechanism [45].

### 3.3. Interactions between Dietary Supplement/Food and CBZ

 A total of twenty-one original literatures covering 13 different dietary supplement/food-CBZ interaction studies were recorded in the current review. These included beverages (*n* = 7), food substances (*n* = 3), and dietary supplements (*n* = 3). Alcohol did not affect the pharmacokinetics of CBZ in healthy volunteers but increased the oral bioavailability and decreased the metabolism of CBZ in alcoholics [55]. Alcohol-CBZ combination also had an additive neurotoxicity in animals [54]. Another beverage Coca-Cola increased the oral bioavailability of CBZ in a clinical study which may be due to the enhanced dissolution of CBZ by its acidity [60]. Caffeine decreased the oral bioavailability as well as the antiepileptic efficacy of CBZ in human and animal studies, respectively [57–59]. Four juices, namely, grapefruit juice, kinnow juice, pomegranate juice, and star fruit juice, were demonstrated to increase the oral bioavailability of CBZ through inhibiting enteric CYP3A4 activity [62, 66, 71, 74] though an *ex vivo* study suggested that pomegranate juice might induce enteric CYP3A4 due to the decreased intestinal permeation of CBZ [72]. Pharmacokinetic interactions between food substances and CBZ were recorded: butter increased while soy bean decreased the oral bioavailability of CBZ in animal study. The former might improve dissolution of CBZ while the later might decrease the gastric emptying and enhance the metabolism of CBZ [56, 73]. Although honey was shown to decrease the oral bioavailability of CBZ in rabbits, it had no effect on the pharmacokinetic parameters of CBZ in human [63–65]. As regards the dietary supplement-CBZ interactions, folinic acid did not alter the plasma level of CBZ in rats [61] while nicotinamide increased CBZ plasma level and decreased its clearance in two children with epilepsy [70]. Melatonin did not interact with CBZ pharmacokinetically but potentiated the antiepileptic activity of CBZ in both animal and human studies [67–69].

## 4. Discussion

Patients on antiepileptic therapy are usually on a long-term basis. Several antiepileptic drugs require therapeutic drug monitoring and are prone to drug interactions which may lead to serious consequences. CBZ is one of the antiepileptic drugs that are on the “watch-list”. With the increased popularity of herbal products as well as dietary supplement, prescribers may need to be aware of the potential herb-drug or food-drug interactions when prescribing and monitoring CBZ therapy. In this study, we had conducted a systematic review and summarized the up-to-date evidence of the interactions between CBZ and herbal products/food/dietary supplements that have been reported in primary literature. 

In order to achieve a comprehensive literature search, a total of eleven databases were searched. These included two conventional databases (EMBASE and MEDLINE), five other English databases (AMED, CINAHL Plus, Cochrane Database of Systematic Reviews, CENTRAL, and SciFinder Scholar) four Chinese databases. We had also consulted some relative tertiary literatures including Stockley's Herbal Medicines Interactions and Natural Medicines Comprehensive Database in case of any additional information. The keywords used for the search were optimized and refined in an attempt to include most of the relevant literatures (Table 1). We suggest that this search strategy could be applied on the search of other drugs—herb/food/dietary supplement interactions by substituting the drug name. It is interesting to note that, although a total of 100 clinical trials or case reports involving the concurrent use of TCMs with CBZ were identified from the Chinese databases, more than 90 of them focus on the beneficial effects or the antagonism of the side effects of CBZ of such combinational use and fallout from our inclusion criteria.

There were altogether 33 different herbal products/food/dietary supplements identified from literature in which their effects on CBZ were studied. These included 17 TCMs, 3 other herbs/botanicals, 10 foods, and 3 dietary supplements. The large number of studies involving TCM-drug interactions implies that TCM warrants special attention when coadministered with CBZ. However, the nonstandardized naming and multiple constituents of TCMs often confuse prescribers when anticipating such interaction. After extracting the herbal names from the original articles, we standardized the herbal names in Latin according to the Chinese Pharmacopoeia 2010 (Table 2). In order to raise the prescribers' awareness to the different names of the TCM products, we also provide the synonyms of the included TCMs in Table 4. For herbal formulae, their composition and content were also listed (Table 5).

No fatal or severe interactions between CBZ and herbal products/food/dietary supplement were found from the literature search. Majority of the studied interactions were pharmacokinetic-based, where the oral bioavailability or plasma level of CBZ was significantly altered by the natural products (Table 6). Twelve natural products/food, elevated the oral bioavailability/plasma level of CBZ with six of them demonstrating clinical evidence, namely, piperine, alcohol, Coca-Cola, grapefruit juice, kinnow juice, and nicotinamide (highlighted in bold in Table 6). In most cases the authors suggested that the increase in plasma CBZ concentrations was due to the inhibition of CYP3A4-mediated metabolism of CBZ by these natural products. Since CBZ has a narrow therapeutic index, and the side effects of CBZ are concentration-dependent, the increment of CBZ plasma level may result in serious adverse effects such as diplopia and nystagmus [75]. Therefore, it is advised to avoid the consumption of the food/herbal products which could elevate the CBZ plasma level as listed in Table 6. 

On the other hand, nine natural products diminished the oral bioavailability/plasma level of CBZ significantly with four of them having clinical evidences: Ispaghula husk, Xiao-yao-san, Jia-wei-xiao-yao san, and caffeine (Table 4). Multiple mechanisms may contribute to the decrease of CBZ plasma level by these natural products, one of which is the increase in the metabolism of CBZ by induction of CYP3A4 (by Jia-wei-xiao-yao-san, ginkgo biloba, and soybean) and mixed function oxidase (by caffeine) activity. Ispaghula husk, septilin, soybean and Xiao-cha-hu-tang reduced the plasma level of CBZ by affecting its gastric absorption. Coadministration of CBZ with herbal products/food which are enzyme inducers entails the possibility of a clinically significant drug interaction. The reduction of CBZ plasma level may imply that less CBZ is present in the target site to assert its antiepileptic activity; a worsened seizure control may follow unless the dosage of CBZ is adjusted accordingly [76]. Since enzyme induction is a reversible phenomenon, particular caution is required when an enzyme-inducing agent is discontinued because the serum concentration of concurrently administered CBZ may rebound to potentially toxic levels. Patients are therefore not recommended to take the food/herbal products which could decrease the CBZ plasma level listed in Table 6. 

Furthermore, CBZ has poor water solubility; consequently, its absorption time and extent are thus easily affected by coadministration of substances that may alter gastric conditions. For example, Xiao-cha-hu-tang and Xiao-qing-long-tang delayed the time for CBZ to reach peak plasma concentration by decreasing the gastric emptying rate. By improving the dissolution of CBZ, Paeoniae Radix allowed faster absorption while butter and Platycodonis Radix increased the extent absorption of CBZ. 

Any changes of the plasma level of CBZ-10,11 epoxide caused by the simultaneous administration of herbal products/food/dietary supplements with CBZ should also be noted. Formed through the CYP3A4-mediated metabolism in intestine and liver, CBZ-10,11 epoxide is the principle metabolite of CBZ which is pharmacologically active and may contribute to the toxicities of CBZ [77]. Neurotoxic symptoms including ataxia, dizziness, nausea, and diplopia had been observed in patients in which lamotrigine or loxapine was added to CBZ therapy, with elevated blood levels of CBZ-10,11-epoxide [78, 79]. *Polygonum cuspidatum*, a widely used TCM indicated for menstrual and postpartum difficulties, traumatic burns, and acute viral hepatitis, was shown to increase CBZ and CBZ-10,11-epoxide levels in plasma, brain, liver, and kidney in an animal study [49]. On the other hand, Xiao-cha-hu-tang decreased the oral bioavailability of CBZ-10,11-epoxide in rats [51]. Although there was no clinical evidences of these two TCMs causing an elevated/decreased plasma level of CBZ-10,11-epoxide or showing their linkage to pharmacodynamic outcome, it is rational to pay necessary cautions and avoid their combinational use with CBZ.

Compared to pharmacokinetic interactions, there were fewer studies reporting pharmacodynamic-based interactions between herbal products/food/dietary supplements and CBZ. Pharmacodynamic interaction refers to the alteration of efficacy (antiepileptic activity) and/or the adverse effects of CBZ in the presence of natural products. Melatonin and *Acorus calamus* Linn. potentiated the anticonvulsant activity of CBZ but had no effect on the plasma levels of CBZ in animal studies so such interactions are mainly pharmacodynamic-based. Despite the apparent efficacy-boosting effect, it is best to avoid the use of melatonin or *Acorus calamus* Linn. with CBZ until there further clinical evidence on the safe usage of such combination. Caffeine, on the other hand, decreased the plasma level of CBZ in human while decreased the antiepileptic efficacy of CBZ in mouse. Although there are no clinical studies on whether the antiepileptic activity of CBZ is influenced by caffeine, advice should be given to patients on CBZ therapy not to take caffeine. Caution should also be paid for beverage containing alcohol. Alcohol was demonstrated to have additive neurotoxicity with CBZ in mouse, including a potentiated motor incoordination and loss of righting reflex. Together with the fact that alcohol caused an increase in the oral bioavailability of CBZ in alcoholics, it is advised not to consume any alcohol while patients take CBZ. In a randomized double-blinded control trial, Xiao-yao-san increased the incidence of CBZ-related side effects including dizziness, blurred vision, skin rash, and nausea in patients with major depression or bipolar disorder. Though the mechanism is unknown, patients should be warned about the potential risks when taking this TCM with CBZ.

In this study, the documented evidence of interactions between CBZ and herbal products/food/dietary supplements was systematically reviewed from the published literature. The intention of this review was to provide guidance to assist healthcare professionals in identifying patients taking CBZ that are more susceptible to these interactions and make proper actions. A total of 33 unique herbal products/dietary supplement/food-CBZ interacting pairs were identified from this review. Considering the popularity and frequent usage of both CBZ (as first-line epilepsy regimen) and herbal products/food/dietary supplements, the number of studied interactions is considerably small. More evidence and reports are needed from research studies and, preferably, from adverse report system in clinical setting. Of course, the importance of therapeutic drug monitoring of CBZ is again emphasized while most pairs of natural products-CBZ interactions remained unknown. On the other hand, the amount of documented CBZ-herbal products/food/dietary supplements interactions might be underreported in this review due to several limitations, including publication bias and language restrictions. We had attempted to reduce language bias by including four evidence-based Chinese databases. However, the evidence regarding complementary alternative medicine or folk therapies, which were published in other languages (e.g., Japanese, Indian, and French), might be missing. Another limitation of this review was that it included all relevant information identified in the literature, regardless of the evidence types or quality of the studies. Such arrangement aimed to gather as much useful information regarding studies on interactions between CBZ and the natural products. Although species differences existed, human pharmacokinetic parameters and pharmacodynamic behavior could be successfully extrapolated from animal studies [80, 81]. Therefore, the data from animal studies are considered to be valuable, and hence the results should not be neglected.

## 5. Conclusion

This review provides a structured summary of the evidence of the documented interactions between CBZ and herbal products/food/dietary supplements. These findings should be helpful for healthcare professionals to identify potential herb-drug and food-drug interactions while prescribing CBZ and would also facilitate them to communicate these documented interactions to their patients, thus preventing potential adverse events and improving patients' therapeutic outcomes.

## Figures and Tables

**Table 1:** Keyword and MeSH search terms for herbs, food, and dietary supplements.

Keywords	MeSH terms
(i) alter∗ medic∗ (ii) botanical.tw. (iii) (chinese adj (herb$ or drug$ or formul$ or plant$ or presri$ or remed$ or materia medica)).ab,ti,ot. (iv) (drug∗ and chines∗ and herb∗).mp. (v) (herb or herbs or herbal).tw. (vi) herbal remed$.tw. (vii) ((herb$ or drug$ or formul$ or plant$ or presri$ or remed$ or materia medica) adj chinese).ab,ti,ot (viii) integrative medicin$.ab,ti,ot. (ix) Nutrition$ supplement or diet$ supplement.mp. (x) (phytodrug$ or phyto-drug$ or phytopharmaceutical$).tw. (xi) (plant∗ and extract∗).mp. (xii) (plant∗ and medic∗).mp. (xiii) (TCM or CHM).tw. (xiv) (tradition∗ and chines∗ and medic∗).mp. (xv) traditional chinese.tw.	(i) exp Chinese drug (ii) exp Chinese herb (iii) exp Chinese medicine (iv) exp diet supplementation (v) exp drugs, Chinese Herbal (vi) exp food (vii) exp food drug interaction (viii) exp herbal medicine (ix) exp herbaceous agent (x) exp medicine, Ayurvedic (xi) exp medicine, east asian traditional (xii) exp medicine, Chinese traditional (xiii) exp medicine, kampo (xiv) exp medicine, Korean traditional (xv) exp medicine, mongolian traditional (xvi) exp medicine, Oriental Traditional (xvii) exp medicine, tibetan traditional (xviii) exp phytotherapy (xix) exp plant extract (xx) exp Plants, Medicinal (xxi) exp shamanism

**Table 2:** Summary of the included* in vitro*, animal, and clinical studies on interactions between carbamazepine and herbal products, dietary supplement, and food.

Types of herbal product*	Herbal products	Study type	Subject/model (number)	Study design	Outcome measures	Effect	Mechanism	References
TCM	*Acorus calamus* Linn.	Animal	Male Wistar rats with pentylenetetrazole-induced seizure model (6 in each group)	Randomized controlled study	PK and PD parameters	No effect on plasma level of CBZ Additive antiepileptic activity	Increased GABAergic activity	Katyal et al. 2012 [35]

TCM	Berberine (active compound in Coptidis rhizome)	Animal	Male Wistar rats (5 in each group)	Randomized parallel design	PK parameters	No effect on pharmacokinetic parameters of CBZ or CBZ 10,11-epoxide	Did not affect* in vivo* intestinal or hepatic CYP3A activity	Qiu et al. 2009 [36]

TCM	*Cardiospermum halicacabum* Linn.	Animal	Male Wistar rats (10 in each group)	Randomized crossover design	PK and PD parameters	No significant effect on CBZ plasma level No change in drug-related toxicity (including general behavior, liver function, haematological parameters, and kidney function)	N.D.	Thabrew et al. 2004 [37]

TCM	*Cassia auriculata* Linn.	Animal	Male Wistar rats (10 in each group)	Randomized crossover design	PK and PD parameters	Increased plasma level of CBZ No change in drug-related toxicity (including general behavior, liver function, haematological parameters and kidney function)	N.D.	Thabrew et al. 2004 [37]

TCM	Chai-hu-jia-long-gu-mu-li- tang	Animal	Wistar rats (5-6 in each group)	Randomized parallel design	PK parameters	No effect on pharmacokinetic parameters or protein binding of CBZ or CBZ 10,11-epoxide	Did not alter *in vivo* CYP3A activity	Ohnishi et al. 2001 [38]

TCM	Ginsenoside (active compound in *ginseng*)	*In vitro *	Human liver microsomes (3 in each group)	N/A	PK parameters	Increased CBZ metabolism	Activated CYP3A4 activity by interacting with CBZ in the active site	Haop et al. 2008 [39]

TCM	*Ginkgo biloba *	Animal	Rats (6 in each group)	Randomized parallel design	PK parameters	Decreased bioavailability and increased rate of elimination of CBZ	N.D.	Chandra et al. 2009 [40]

TCM	Hu-gan-ning pian	Animal	Male Sprague-Dawley rats (7 in each group)	Randomized parallel design	PK parameters	Decreased bioavailability No effect on *C*_max⁡_, *t*_1/2_, *t*_max⁡_, CL, and elimination *K* of CBZ	Decreased absorption but not metabolism of CBZ	Zheng et al. 2009 [41]

TCM	*Hypericum perforatum *Linn.	Human	Healthy subjects (8)	Open label study	PK parameters	No effect on PK parameters of CBZ	Autoinduction or greater clearance by CBZ	Burstein et al. 2000 [42]

HP	Ispaghula Husk (Psyllium)	Human	Healthy male volunteer (4)	Open label study	PK parameters	Decreased bioavailability by reducing absorption and plasma levels of CBZ	Decreased amount of biological fluid in GI tract and thereby reduced dissolution rate of CBZ Also adsorb CBZ onto their surfaces	Etman 1995 [43]

TCM	Jia-wei-xiao-yao-san	Human	Patients with major depression or bipolar disorder (61)	Randomized double-blinded control trial	PK parameters	Decreased plasma level of CBZ	Increased metabolism of CBZ by inducing CYP3A	Zhang et al. 2007 [44]

HP	Mentat	Animal	New Zealand white rabbits (8 in each group)	Randomized parallel design	PK parameters	Increased bioavailability of CBZ	N.D.	Tripathi et al. 2000 [45]

TCM	Paeoniae Radix	Animal	Male Sprague-Dawley rats (6 in each group)	Randomized parallel design	PK parameters	Decreased *T*_max⁡_ of CBZ Decreased protein binding rate of CBZ No effect on AUC, *C*_max⁡_, *t*_1/2_, CL, and F of CBZ	Improved dissolution of CBZ N.D.	Chen et al. 2002 [46]

TCM	Piperine (active compound in *Piper longum* Linn.)	Human	Patients with epilepsy (10 in each group)	Open label, crossover study	PK parameters	Increased bioavailability of CBZ Increased elimination rate and decreased elimination *t*_1/2_	Decreased metabolism/ elimination and/or increased absorption of CBZ	Pattanaik et al. 2009 [47]

TCM	Platycodonis Radix	Animal	Rabbits (4 in each group)	Randomized parallel design	PK parameters	Increased plasma level of CBZ	Improve CBZ absorption by increasing its solubility and stimulating bile secretion	Liu and Wei 2008 [48]

TCM	*Polygonum cuspidatum *	Animal	Male Sprague-Dawley rats (6 in each group)	Randomized crossover design	PK parameters	Increased level of CBZ and CBZ 10,11-epoxide in plasma, brain, liver, and kidney Decreased formation rate of CBZ 10,11-epoxide	Inhibited CYP3A in intestine and MRP2 in the kidney	Chi et al. 2012 [49]

HP	Septilin	Animal	Male rabbits (8 in each group)	Randomized crossover study	PK parameters	Decreased absorption of CBZ	Affected gastric emptying time or intestinal transit time	Garg et al. 1998 [50]

TCM	Xiao-cha-hu-tang	Animal	Female Sprague-Dawley rats (4 in each group)	Randomized parallel design	PK parameters	Increased *T*_max⁡_, decreased *C*_max⁡_ of CBZ and AUC of CBZ 10,11-epoxide No effect on *t*_1/2_, and MRT_0–*∞*_ of CBZ	Decreased GI absorption of CBZ by decreasing gastric emptying rate	Ohnishi et al. 2002 [51]

TCM	Xiao-qing-long-tang	Animal	Male Wistar rats (4–6 in each group)	Randomized parallel design	PK parameters	Increased *T*_max⁡_, elimination *K* of CBZ and decreased *t*_1/2_ MRT_0–*∞*_ of CBZ No effect on *C*_max⁡_ and AUC of CBZ and CBZ 10,11-epoxide	Decreased gastric emptying rate and accelerated metabolism of CBZ	Ohnishi et al. 1999 [52]

TCM	Xiao-yao-san	Human	Patients with major depression or bipolar disorder	Randomized double-blinded control trial	PK and PD parameters	Decreased plasma level of CBZ and increased incidence of dizziness, blurred vision, skin rash, and nausea	N.D.	Li et al. 2005 [53]

*Types of herbal product: traditional Chinese medicines (TCM)/other herbal products (HP); N.D.: not determined by authors; N/A: not applicable.

**Table 3:** Summary of the included *in vitro*, animal and clinical studies on interactions between carbamazepine and dietary supplement/food.

Dietary supplement (DS)/food	Dietary products	Study type	Subject/model (number)	Study design	Outcome measures	Effect	Mechanism	References
Food	Alcohol	Animal	Male CD-1 mice (10 in each group)	Randomized parallel design	PD parameters	Additive neurotoxicity (ethanol-induced motor incoordination and loss of righting reflex potentiated )	Nonadenosinergic action	Dar et al. 1989 [54]

Food	Alcohol	Human	Healthy volunteers (8)	Open label crossover study	PK parameters	No effect on pharmacokinetics of CBZ	Low ethanol level in subjects	Sternebring et al. 1992 [55]

Food	Alcohol	Human	Alcoholics (7)	Open label crossover study	PK parameters	Increased AUC_0–12 h_ of CBZ and decreased AUC_0–12 h_ of CBZ 10,11-epoxide	Acute inhibition of CBZ metabolism and/or accelerated CBZ metabolism in abstinence phase due to enzyme induction by previous ethanol abuse	Sternebring et al. 1992 [55]

Food	Butter	Animal	New Zealand white rabbit (8 in each group)	Crossover study	PK parameters	Increased bioavailability of CBZ	Improved solubility and dissolution of poorly soluble CBZ	Sidhu et al. 2004 [56]

Food	Caffeine	Human	Healthy male volunteers (6)	Open label crossover study	PK parameters	Decreased bioavailability and increased *V*_*d*_ of CBZ	Involving metabolism by mixed function oxidase	Vaz et al. 1998 [57]

Food	Caffeine	Animal	Albino Swiss male mice with maximal electroshock seizure model (7 in each group)	Randomized controlled parallel study	PK and PD parameters	Acute caffeine decreased antiepileptic efficacy of CBZ but had no effect on plasma level of CBZ	N.D.	Czuczwar et al. 1990 [58]

Food	Caffeine	Animal	Swiss male mice with maximal electroshock seizure model (8 in each group)	Randomized controlled study	PK and PD parameters	Chronic caffeine dose-dependently decreased anti-epileptic efficacy of CBZ but had no effect on plasma level of CBZ	May induce changes in neurotransmitter system causing sensitization effect	Gasior et al. 1996 [59]

Food	Coca-Cola	Human	Healthy male volunteers (10)	Randomized two-way crossover design	PK parameters	Increased bioavailability of CBZ; no change in elimination *t*_1/2_	Enhanced dissolution of CBZ by its acidity	Malhotra et al. 2002 [60]

DS	Folinic acid	Animal	Male Sprague-Dawley rats (4 in each group)	Randomized parallel controlled design	PK parameters	No effect on plasma or brain level of CBZ	N.D.	Simth and Carl 1982 [61]

Food	Grapefruit juice	Human	Patients with epilepsy (10)	Randomized crossover study	PK parameters	Increased bioavailability of CBZ	Inhibited CYP3A4-mediated intestinal and hepatic metabolism of CBZ	Garg et al. 1998 [62]

Food	Honey	Animal	Angora grey rabbit (6 in each group)	Nonrandomized design	PK parameters	Decreased bioavailability of CBZ	Decreased metabolism of CBZ by inducing CYP enzymes	Koumaravelou et al. 2002 [63]

Food	Honey	Human	Healthy volunteers (10)	Randomized crossover study	PK parameters	Single dose of honey has no effect on pharmacokinetics of CBZ	N.D.	Malhotra et al. 2003 [64]

Food	Honey	Human	Healthy male volunteers (12)	Open label crossover study	PK parameters	Multiple doses of honey have no effect on pharmacokinetics of CBZ	Flavanoids in honey may not affect human CYP3A4 activity	Thomas et al. 2007 [65]

Food	Kinnow Juice	Human	Healthy male volunteers (9)	Randomized crossover study	PK parameters	Increased bioavailability of CBZ	Inhibited CYP3A activity	Garg et al. 1998 [66]

DS	Melatonin	Animal	Female Swiss mice (12 in each group)	Randomized parallel design	PK and PD parameters	Potentiated the anticonvulsant activity of CBZ but impair long-term memory but no effect on plasma and brain levels of CBZ	Enhanced GABAergic transmission in CNS	Borowicz et al. 1999 [67]

DS	Melatonin	Human	Children with epilepsy (28)	Double-blind randomized control study	PK and PD parameters	Increased glutathione reductase (antioxidant) activity but no effect on plasma level of CBZ and its metabolite	Antagonized CBZ-triggered reactive oxygen species accumulation	Gupta et al. 2004 [68]

DS	Melatonin	Animal	Male Swiss albino mice with maximal electroshock seizure model (7 in each group)	Randomized parallel design	PK and PD parameters	Synergistic anti-epileptic effect but no effect on plasma level of CBZ	N.D.	Gupta et al. 2004 [69]

DS	Nicotinamide	Human	Children with epilepsy (2)	Case report	PK parameters	Increased plasma level of CBZ and decreased clearance of CBZ	N.D.	Said et al. 1989 [70]

Food	Pomegranate juice	Animal	Male Wistar rats (5-6 in each group)	Randomized parallel design	PK parameters	Increased *C*_max⁡_ and AUC of CBZ; no change in elimination *t*_1/2_ and AUC ratio of CBZ 10,11-epoxide to CBZ	Inhibited enteric but not hepatic CYP3A activity	Hidaka et al. 2005 [71]

Food	Pomegranate juice	*Ex vivo *	Male Wistar rats (3 in each group)	*In vitro *everted and noneverted sac method	PK parameters	Decreased intestinal transport of CBZ	Induced enteric CYP3A4	Adukondalu et al. 2010 [72]

Food	Soybean	Animal	Albino Wistar rats (6 in each group)	Randomized parallel design	PK parameters	Decreased bioavailability of CBZ, increased plasma clearance and *V*_*d*_ of CBZ	Decreased gastric emptying and enhanced elimination of CBZ	Singh and Asad 2010 [73]

Food	Star Fruit Juice	Animal	Male Wistar rats (6 in each group)	Randomized parallel design	PK parameters	Increased *C*_max⁡_ and AUC of CBZ; no change in elimination *t*_1/2_ and AUC ratio of CBZ 10,11-epoxide to CBZ	Inhibited enteric but not hepatic CYP3A activity	Hidaka et al. 2006 [74]

N.D.: not determined by authors.

**Table 4:** Synonyms of the included TCM products.

Herbal products	Synonyms
*Acorus calamus* Linn.	Sweet flag, Zhang-chang-pu
*Cardiospermum halicacabum* Linn.	Ballon vine, Winter cherry, Heartseed, Dao-di-ning
*Cassia auriculata* Linn.	Avaram, Senna auriculata, Tanner's Cassia, Er-ye-fan-xie
*Hypericum perforatum* Linn.	St John's wort
Paeoniae Radix	Peony, Shao-yao
Platycodonis Radix	Jie geng, Platycodon Root, Balloon flower
*Polygonum cuspidatum *	Japanese knotweed, Hu-zhang

**Table 5:** Composition of individual herbs in the included herbal formulae.

Herbal formula	Other name	Herbs	Content
Chai-hu-jia-long-gu-mu-li-tang	Saiko-ka-ryukostsu-borei-to	Bupleuri Radix Pinelliae Tuber Cinnamomi Cortex Hoelen Scutellariae Radix Zizyphi Fructus Ginseng Radix Ostreae testa Fossilia Ossis Mastodi Zingiberis Rhizoma	5 parts 4 parts 3 parts 3 parts 2.5 parts 2.5 parts 2.5 parts 2.5 parts 2.5 parts 1 part

Hu-gan-ning pian	Huganning tablet	Sedi Herba Polygoni Cuspidati Rhizoma et Radix Salviae Miltiorrhizae Radix et Rhizoma Ganoderma	850 g 500 g 250 g 200 g

Jia-wei-xiao-yao-san	Free and easy wanderer plus	Bupleuri Radix Scutellariae Radix Zingiberis Rhizoma Angelicae sinensis Radix Zizyphi Fructust Moutan Cortex Paeoniae Radix Alba Atractylodis Macrocephalae Rhizoma Poria Menthae Haplocalycis Herba Glycyrrhizae Radix	12.5% 12.5% 11.2% 9.7% 9.7% 9.7% 9.7% 8.3% 6.9% 5.6% 4.2%

Mentat	BR 16A	*Bacopa monnieri* Linn. *Centella asiatica* Linn. *Withania somnifera* Linn. *Evolvulus alsinoides* Linn. *Nardostachys jatamansi* Linn. *Acorus calamus* Linn. *Celastrus paniculatus* Linn. *Zingiber officinale* Linn. Valeriana wallichii Prunus amygdalus *Orchis mascula* Linn. *Syzygium aromaticum *Linn. Mukta pishti	Not known

Xiao-cha-hu-tang	Sho-saiko-to	Bupleuri Radix Pinelliae Tuber Scutellariae Radix Zizyphi Fructus Ginseng Radix Glycyrrhizae Radix Zingiberis Rhizoma	7 parts 5 parts 3 parts 3 parts 3 parts 2 parts 1 part

Xiao-qing-long-tang	Sho-seiryu-to extract	Pinelliae Tuber Glycyrrhizae Radix Cinnamomi Cortex Schisandrae Fructus Asiasari Radix Paeoniae Radix Ephedrae Hebra Zingiberis Siccatum Rhizoma	6 parts 3 parts 3 parts 3 parts 3 parts 3 parts 3 parts 3 parts

Xiao-yao-san	Free and easy wanderer	Bupleuri Radix Angelicae sinensis Radix Paeoniae Radix Alba Atractylodis Macrocephalae Rhizoma Poria Zingiberis Rhizoma Glycyrrhizae Radix Menthae Haplocalycis Herba	2 parts 2 parts 2 parts 2 parts 2 parts 2 parts 1 part 1 part

**Table:** (a)

Pharmacokinetic interactions with CBZ		
Oral bioavailability/plasma level of CBZ		
Increased	Decreased	No effect
**Piperine** Mentat *Polygonum cuspidatum* Butter **Grapefruit juice** Platycodonis Radix Pomegranate juice Star fruit juice **Kinnow juice** Alcohol* **Coca-cola** **Nicotinamide**	Septilin *Ginkgo biloba* Hu-gan-ning pian **Ispaghula husk** *Cassia auriculata *Linn. Hu-gan-ning pian Xiao-cha-hu-tang **Xiao-yao-san** **Jia-wei-xiao-yao-san** Soybean **Caffeine**	***Hypericum perforatum *Linn.** Paeoniae Radix *Cardiospermum halicacabum *Linn. Berberine Xiao-qing-long-tang Chai-hu-jia-long-gu-mu-li-tang *Acorus calamus* Linn. **Honey** **Melatonin** Folinic acid

*In alcoholics, not healthy volunteers; study type: human study (**bold**), animal study (regular).

**Table:** (b)

Pharmacodynamic interactions with CBZ					
Antiepileptic efficacy of CBZ			Side effects related to CBZ		
Potentiation	Inhibition	No effect	Potentiation	Reduction	No effect
*Acorus calamus* Linn. Melatonin	Caffeine		**Xiao-yao-san** Melatonin Alcohol		*Cassia auriculata *Linn. *Cardiospermum halicacabum *Linn.

Study type: human study (**bold**), animal study (regular).
